# Multimodal Navigation System for Visually Impaired Users Using Environmental Perception and Vision-Language Models †

**DOI:** 10.3390/s26103045

**Published:** 2026-05-12

**Authors:** Huei-Yung Lin, Yu-Hsiang Fan, Chin-Chen Chang

**Affiliations:** 1 Department of Computer Science and Information Engineering, National Taipei University of Technology, Taipei 10608, Taiwan; lin@ntut.edu.tw (H.-Y.L.);; 2 Department of Computer Science and Information Engineering, National United University, Miaoli 360302, Taiwan

**Keywords:** assistive navigation, multimodal interaction, large language models, vision-language models

## Abstract

Visually impaired users face significant challenges in navigating complex indoor environments due to limited spatial awareness and lack of real-time semantic guidance. This paper proposes a multimodal navigation system integrating environmental perception with vision-language models (VLMs). It provides context-aware and explainable guidance without requiring additional infrastructure. The proposed system combines RTAB-Map for localization, YOLO-World for open-vocabulary object detection, and a lightweight language model for semantic reasoning and natural language interaction. To evaluate our system, experiments are conducted using the RePOPE benchmark to assess hallucination in vision-language understanding. Real-world indoor navigation experiments are also performed. The results show that integrating perception with language-based reasoning improves precision by up to 2.29% and consistently enhances F1-score compared to baseline VLM approaches. Real-world experiments further demonstrate reliable navigation performance, including multi-floor path planning and obstacle-aware guidance. Hence, the proposed system effectively enhances spatial understanding and reduces hallucination, providing a practical and scalable solution for assistive navigation.

## 1. Introduction

Visually impaired users around the world encounter considerable difficulties when navigating complex indoor environments. In these environments, basic directional cues are insufficient, and more detailed semantic information becomes essential. Conventional assistive technologies, such as white canes and guide dogs, primarily support obstacle avoidance but lack the ability to deliver advanced spatial understanding and contextual understanding. Although integrated sensor-based systems have been proposed [1,2,3], they often suffer from inaccurate localization and limited semantic interpretation. Moreover, their largely machine-centered interaction approaches may reduce user engagement and weaken trust. The rapid advancement of large language models (LLMs) and vision-language models (VLMs) has significantly enhanced cross-modal perception and semantic reasoning capabilities of machines. These developments open new opportunities for building semantic-driven navigation systems specifically designed to better support visually impaired users.

To address these challenges, we move beyond traditional one-way control systems by adopting a human-centric, machine-assisted collaborative navigation approach. The proposed system integrates voice input, real-time visual perception, and language understanding. It provides semantic-rich descriptions of the environment and clear action suggestions. This design allows safer and more efficient user-driven navigation aligned with human-centered principles.

Traditional navigation systems for visually impaired users often depend on expensive and non-portable infrastructure, such as radio frequency identification (RFID) [4,5], Wi-Fi [6], and ultra-wideband (UWB) [7]. Although simultaneous localization and mapping (SLAM) methods improve localization accuracy [8,9,10], it is still difficult to achieve stable semantic path planning using only visual sensors. In addition, several systems lack natural language interaction. To solve these problems, researchers have proposed topological semantic maps [11,12,13]. When combined with LLMs, these maps further improve semantic reasoning and natural language interaction [14]. Existing systems fail to jointly address semantic reasoning, real-time perception, and explainable human-centered guidance.

In this paper, we present a collaborative indoor navigation system for visually impaired users. The proposed system integrates voice input, real-time images, and semantic maps. It provides semantic path suggestions and clear descriptions of the environment to support user decision-making. Unlike traditional machine-centered systems, the proposed approach emphasizes human-dominant interaction. It uses semantic explanations and visual support to improve spatial understanding of indoor scenes. Our system uses real-time appearance-based mapping (RTAB-Map) [15] to build semantic maps. We apply YOLO-World [16] for object detection and use GPT-4o-mini, a lightweight vision-language model capable of multimodal reasoning, as the core model for language and visual reasoning. Our system also integrates open street map area graph (OSMAG) topological semantic maps [12] and natural language generation functions. In addition, it includes speech recognition and speech synthesis modules. These modules allow natural voice interaction between the user and our system. Visually impaired users can make decisions based on contextual understanding. By tightly combining language and vision, the proposed system provides clear and explanatory guidance. This design improves human–robot collaboration and enhances user trust, overcoming the limitations of traditional navigation systems.

The main contributions of this paper are summarized as follows:

- We propose a multimodal navigation framework that integrates real-time perception, topological semantic mapping, and vision-language reasoning to provide context-aware guidance for visually impaired users.
- Unlike conventional vision-based systems that rely solely on end-to-end VLM inference, our approach introduces perception-grounded reasoning by incorporating YOLO-based object detection to reduce hallucination and improve environmental reliability.
- We design a semantic path planning and explanation mechanism that transforms structured topological paths into interpretable natural language instructions, enhancing user understanding and trust.
- We validate the proposed system through both benchmark-based evaluation and real-world navigation experiments, demonstrating improved accuracy, robustness, and practical feasibility compared to baseline approaches.

The remainder of this paper is organized as follows: Section 2 reviews related works, while Section 3 introduces the proposed methodology. Section 4 describes the results. A discussion of findings and potential future directions is provided in Section 5, followed by concluding remarks in Section 6.

## 2. Related Works

Navigation assistance for visually impaired users has been studied in several ways to support safe and effective movement. These methods can be categorized into three main types: traditional assistive tools, infrastructure-based navigation systems, and vision-based systems that rely on real-time sensing and advanced computational techniques.

Traditional tools, such as white canes, used tactile feedback to detect obstacles and changes in the environment. Guide dogs provided direction and helped avoid obstacles, but they were expensive and required long-term training. Although these methods were helpful, they depended heavily on the user’s experience and spatial memory. They also cannot provide real-time semantic information in unfamiliar environments. Infrastructure-based systems used technologies such as RFID, Wi-Fi, and UWB for indoor localization. Although they can improve positioning accuracy, they require multiple sensors or tags to be installed in the environment, leading to high installation and maintenance costs. They were also sensitive to signal interference and were difficult to relocate or deploy in new environments.

Advances in computer vision and wearable devices have increased interest in vision-based navigation systems. Labbé and Michaud [15] extended the capabilities of real-time appearance-based mapping (RTAB-Map) to compare visual and light detection and ranging (LiDAR)-based SLAM configurations for autonomous robot navigation. Their system supported RGB-D, stereo, and 3D LiDAR inputs and incrementally constructed both topological and metric maps for navigation and localization. However, RTAB-Map may suffer from high memory consumption during long-term operation. Ou et al. [9] combined dynamic SLAM with panoptic segmentation. Their system can distinguish between static structures and moving objects and provide real-time voice guidance. Rui et al. [8] integrated oriented fast and rotated brief SLAM [17] with YOLO-based object detection in a wearable device. The system uses depth sensors and multimodal feedback, such as vibration and speech, to assist navigation. Bamdad et al. [10] reviewed various SLAM-based assistive systems. They highlighted the need for better adaptation to dynamic environments, improved semantic understanding, and more extensive real-world user evaluations.

Although SLAM-based systems improved localization, they often used traditional path planning methods and simple voice prompts. This limited their ability to perform semantic reasoning and support natural interaction. To improve this situation, semantic topological maps have been proposed. These maps organized the environments using functional labels, such as “kitchen” or “corridor.” They also used node-edge structures to represent spatial connections. Li et al. [11] used Google Tango to perform real-time SLAM and semantic labeling. They built semantic topological maps for assistive navigation. Sun et al. [13] proposed a text-driven and unsupervised image segmentation method. This technique extracted semantic regions from architectural floor plans without manual labeling. Feng et al. [12] introduced an OSMAG for mobile robotics. It was a hierarchical topological map that described room connections through areas and gates. Xie and Schwertfeger [14] combined OSMAG with large language models for supporting semantic path reasoning and natural language guidance. However, LLMs struggled with precise metric calculations. Additionally, token constraints limited map complexity, and the models cannot yet guarantee mathematically optimal paths compared to traditional algorithms.

Multimodal models [18,19,20,21,22] have been widely applied in assistive navigation research. These models integrated language, visual, and auditory information. Chen et al. [18] investigated the hallucination issue in large VLMs and introduced external segmentation data to mitigate errors. Their approach improved the accuracy of scene descriptions in wearable assistive systems. Hao et al. [19] employed wearable cameras and audio modules to enhance spatial awareness for multimodal reasoning. Their system also adopted chain-of-thought prompting to improve interpretability. However, it does not incorporate topological maps. VLMs, such as VisionGPT [20], can understand scenes and support natural interaction by processing both text and images. This system further incorporated YOLO-World with prompt-based guidance to generate concise voice descriptions of unusual situations. Zhao et al. [21] introduced a task and benchmark that extended traditional visual question answering to actionable, step-by-step assistance for visually impaired users. Their evaluation was conducted using a visual question answering (VQA) framework. However, existing models still suffered from weak environmental grounding and insufficient fine-grained instructions.

Xu et al. [23] proposed a wearable multimodal navigation system to assist visually impaired users in independent travel. Users interacted through voice commands and received navigation feedback via audio. Their system improved mobility and travel safety through multimodal sensing and interaction. However, its performance depended on positioning signals and visual recognition, which may reduce accuracy in complex environments. Zhang et al. [24] presented a mobile navigation system designed for visually impaired users using multimodal interaction. Their system integrated computer vision, speech interaction, and a large language model to understand surrounding environments and provided conversational navigation guidance. However, their system depended on accurate visual perception and stable mobile computing, which may limit performance in complex environments. Joo et al. [25] proposed a socially intelligent smart cane designed for indoor navigation for visually impaired users. Their system integrated RGB-D sensing, YOLOv8-based object detection, and D* Lite path planning to perceive the environment and generate safe navigation paths. It also incorporated multimodal human–robot interaction, including voice communication and feedback, to support social navigation and user guidance. However, its performance may depend on sensor accuracy and computational resources.

In summary, prior work on assistive navigation can be broadly categorized into traditional tools, infrastructure-based systems, and vision-based approaches. While each category offered specific advantages, they shared common limitations, including insufficient semantic understanding, limited adaptability to dynamic environments, and a lack of explainable human-centered interaction. Recent advances in vision-language models have shown promise in bridging perception and language. However, they often suffered from hallucination and weak environmental grounding. These observations revealed a key research gap. A unified framework was needed to tightly integrate perception, semantic reasoning, and interaction while maintaining robustness and interpretability. The proposed system addressed this gap by combining perception-grounded reasoning with topological semantic mapping to allow context-aware and reliable navigation guidance.

## 3. Methodology

**Figure 1:**
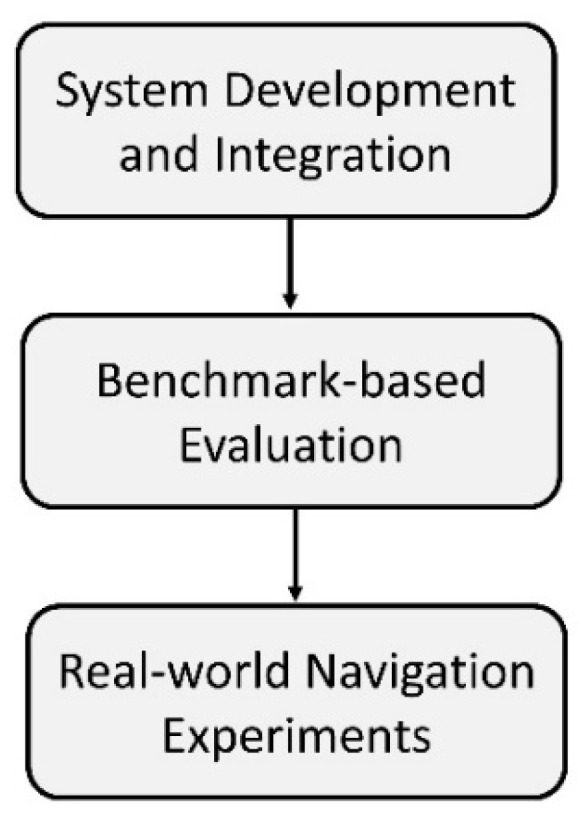
Research workflow of our system.

To provide an overview of the study design, Figure 1 illustrates the overall research workflow, which consists of three main phases: system development and integration, benchmark-based evaluation, and real-world navigation experiments. These phases correspond to the methodological components described in the following subsections.

### 3.1. System Development

**Figure 2:**
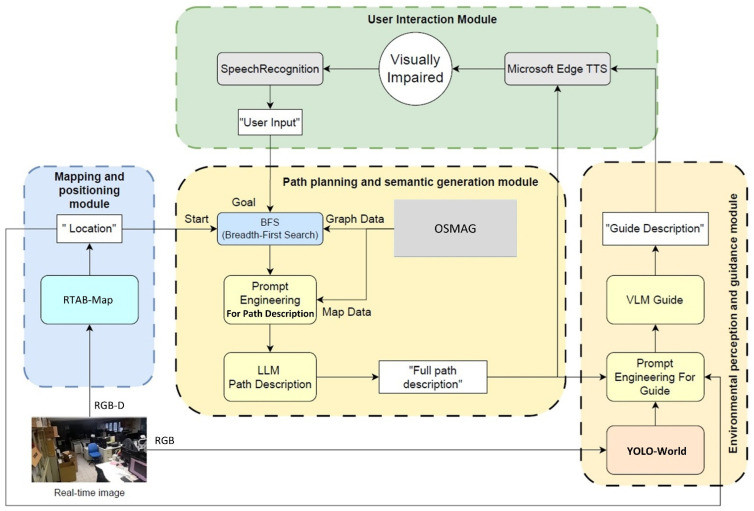
System architecture of the proposed multimodal navigation framework.

We propose a user-centered navigation system combining semantic reasoning with multimodal interaction. The proposed system uses image perception, topological semantic maps, LLMs, and VLMs. The proposed indoor navigation system is designed for visually impaired users. Figure 2 presents the system architecture. It includes four main modules: user interaction, path planning and semantic generation, mapping and positioning, and environment perception and guidance. Our system receives voice commands from the user and integrates real-time perception with LLM-based reasoning to provide clear and meaningful navigation guidance.

In the user interaction module, speech recognition transcribes the user’s voice input into text, which defines the target location. In the mapping and positioning module, we use RTAB-Map for real-time indoor localization and semantic navigation. Meanwhile, a semantic topological map generated by OSMAG is represented as a graph structure. In the path planning module, a breadth-first search (BFS) with semantic constraints computes the path from the current position to the target. The resulting path is then formatted as a prompt for the LLM, which generates a clear, step-by-step semantic route description. In the environment perception and guidance module, real-time scene understanding is performed to enhance guidance accuracy and responsiveness. RGB images are processed by YOLO-World to detect objects such as doors, chairs, and obstacles. The detected visual information and planned semantic path are fed into a multimodal model. It generates context-aware action instructions. Finally, the generated text is converted into speech using Microsoft Edge Text-to-Speech (TTS) and delivered to the user to support safe and goal-directed navigation.

An illustrative example is provided as follows. A user requests navigation to a restroom through the user interaction module. The path planning and semantic generation module identifies the target location and generates a semantic path based on the topological semantic map. Meanwhile, the mapping and positioning module continuously estimates the user’s location to ensure alignment with the planned route. As the user moves, the environment perception and guidance module detects surrounding objects such as doors and corridors and provides real-time feedback. Based on the perceived information, the vision-language model generates step-by-step guidance, such as “walk forward along the corridor and turn right at the door.” If an obstacle is detected, the system dynamically updates the instructions. This example demonstrates how the four modules are integrated to allow real-time, context-aware navigation assistance.

### 3.2. System Integration

#### 3.2.1. Mapping and Positioning

In the mapping and positioning module, we use RTAB-Map as the main SLAM system to support real-time localization and semantic navigation indoors. RTAB-Map is an RGB-D, stereo, and graph-based SLAM approach that detects loop closures and corrects pose errors. It combines image similarity with data from RGB images, depth images, and inertial measurement unit (IMU) sensors. It works well with different sensors and environments. During map building, our system runs RTAB-Map in mapping mode. It collects RGB and depth data saved in the RTAB-Map database format. This format supports visualization and later semantic labeling. Semantic labels are added manually in the RTAB-Map graphical interface. Users can assign labels such as “elevator entrance,” “corridor,” or “classroom” to specific nodes. Each label is linked to a node ID and its point cloud data. This connection supports later semantic region inference and localization queries.

After map building and semantic labeling are finished, our system switches to localization mode. It loads the saved map data and performs real-time localization. The proposed system outputs the current 3D position and pose of the user. Our system also checks nearby semantic nodes around the current position. It uses these nodes to determine the user’s current semantic area. These semantic localization results are sent to the path planning and semantic generation module as the initial input.

#### 3.2.2. Path Planning and Semantic Generation

Traditional navigation systems often provide limited semantic understanding of the environment. Our user-centered indoor navigation system combines topological semantic maps with LLMs. Our system focuses on semantic-based path planning. It uses structured spatial modeling and natural language generation. This design improves the intelligence and clarity of path explanations. It also enhances interactive guidance. This module contains three components:

(1) Topological semantic map construction: To help the proposed system understand spatial connections and semantic meanings, we add semantic labels to traditional topological structures. We follow the concept of OSMAG. In this method, “area” and “gate” are the basic units. We also add semantic attributes to describe indoor spatial relationships. The OSMAG map in our system is drawn manually from the indoor floor plan. It is simplified and enriched with semantic information. This design meets the needs of visually impaired navigation. Each node represents a fundamental unit, referred to as a “way” (area unit). Each node contains up to five semantic attributes:

- level: floor number;
- name: region name;
- OSMAG areaType: type (room or corridor);
- left neighbor/right neighbor: names of adjacent regions;
- up: vertical connections to other floors.

(2) Path planning method: We extend the BFS algorithm with semantic constraints to find paths on the topological semantic map. This component gives the starting node, and voice commands define the target node. BFS finds the shortest sequence of nodes, including floor changes and spatial labels. This sequence is then used by the LLM to generate semantic path instructions. For example, a path from Classroom 1421 to Classroom 1521 can be represented as: {“path”: [“1421”, “14 elevator 1”, “14 stairs 1”, “15 stairs 1”, “15 elevator 1”, “1521”]}.

(3) LLM semantic path generation: This component uses an LLM to convert node sequences into clear, user-friendly navigation descriptions. We design structured prompts to guide the model’s reasoning and text generation. The prompts include the loaded OSMAG map and its structural description. They also include the graph representation with node descriptions and the complete node path sequence. Finally, the prompts provide task instructions, allowing the model to produce natural language guidance.

After receiving this information, the LLM generates a natural language description of the path. The description includes semantic structure, directional cues, and spatial relationships. For example, when navigating from Room 1421 to Room 1521, the proposed system can produce guidance such as:

{Navigation Path: To navigate from Room 1421 to Room 1521, follow these steps:
  (1)  Begin at “Room 1421” on the 14th floor.
  (2)  Head toward “14 elevator 1” and pass by it, as it is out of service.
  (3)  Continue to “14 stairs 1”, which provides access between floors.
  (4)  Take the stairs up to the “15th floor”, arriving at “15 stairs 1”.
  (5)  Proceed toward “15 elevator 1” and pass it, as it is also out of service.
  (6)  Continue forward to reach “Room 1521”.
By following this route, you can reach your destination efficiently while avoiding the broken elevators.}

**Figure 3:**
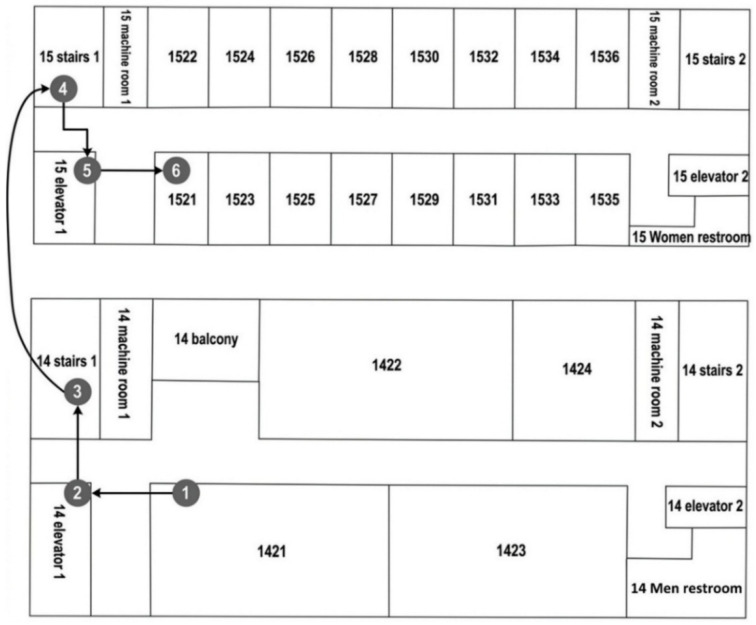
Visualization of path planning results across two floors.

These detailed and semantic descriptions help visually impaired users understand the path more clearly. Users can identify the starting point, the destination, the travel direction, floor changes, and important locations along the route. The generated navigation instructions can be converted into voice prompts for user interaction. Figure 3 illustrates the corresponding navigation path and floor plan. This improves interaction and enhances the interpretability of navigation guidance.

#### 3.2.3. Environmental Perception and Guidance

After path planning, the proposed system needs the environmental perception and guidance module to help visually impaired users move safely in real environments. Our system combines image input with language model reasoning to generate voice prompts. This improves real-time feedback and human-centered interaction. Our system includes two components: the object recognition and the VLM real-time guidance.

(1) Object recognition: To improve real-time perception, we use YOLO-World to detect important objects related to navigation. YOLO-World is based on Ultralytics YOLOv8. It is an open-vocabulary object detection model with zero-shot recognition ability. It can detect several objects using semantic prompts without additional training. We use GPT-4 to generate a list of 25 common obstacles that visually impaired users may encounter. These objects are used as detection targets. Our system identifies key objects such as doors, stairs, chairs, and other obstacles. This helps the proposed system understand changes in the environment and overcome the limitations of traditional static path guidance.

(2) VLM real-time guidance: This component uses a VLM to generate voice guidance. It improves the proposed system’s semantic understanding and interaction with users. The component combines information from the object recognition component and the LLM semantic path generation component. It also uses a task-oriented prompt structure. The prompt includes the current location, the planned path (node sequence), the semantic path description generated by the LLM, and visible object information from the current image, such as doors or obstacles. Based on this information, the VLM generates clear voice messages. For example, it may say: “You are in Room 1421. You will pass Room 1423. Please go straight.” It may also warn the user with messages such as “There is a chair ahead. Please go around it or move carefully,” or “There are stairs ahead. Please go up slowly.” This guidance method provides more contextual information than traditional map coordinates or icon-based navigation. It can also adapt to different environments and better support the needs of visually impaired users.

#### 3.2.4. User Interaction

To provide a natural and simple human–machine interface, our system uses the user interaction module. Visually impaired users can speak their navigation goals and receive voice guidance from the proposed system. This creates a two-way, real-time voice interaction. For voice input, we use the Python package SpeechRecognition (version 3.14.2) and the Google Speech Recognition API as the backend engine. This setup supports several types of microphones and provides stable speech recognition with multiple languages. The spoken commands are quickly converted into text. The text is then used by the language model for semantic understanding and task reasoning. This module mainly handles voice signal capture and speech transcription.

Voice feedback is generated using the Edge-TTS package (version 7.0.2) using the Microsoft Edge Text-to-Speech engine to produce natural and clear speech. Messages generated by other modules are quickly converted into voice prompts and played to the user. Our system also includes a simple fault-tolerance mechanism. If no voice input is detected, the proposed system asks the user to repeat the command and starts listening again. This helps keep the interaction smooth and continuous. With this module, our system supports seamless voice communication. It also reduces the learning difficulty and operating barriers for visually impaired users.

### 3.3. Experimental Setup

**Figure 4:**
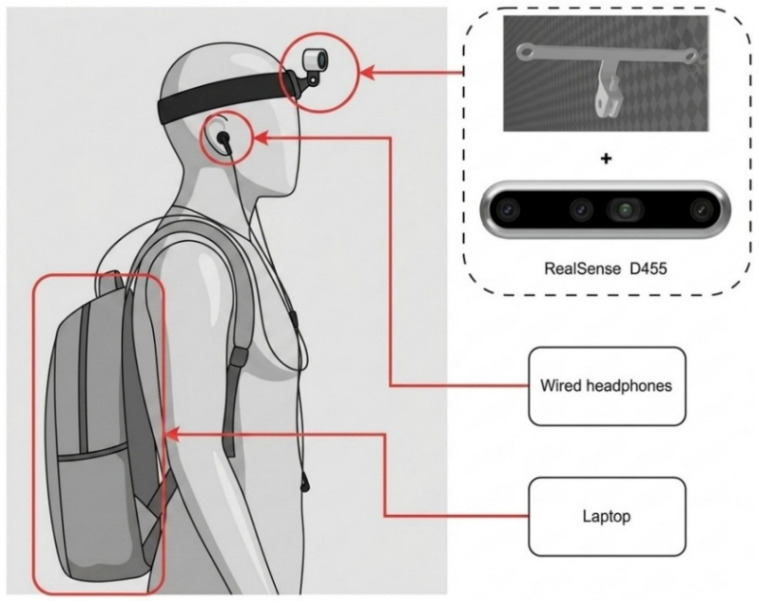
Wearable configuration of the proposed system. From top to bottom: RealSense D455, headphones, and a laptop mounted on the back.

This study focused on system-level validation and did not include visually impaired participants. All experiments were conducted by members of the research team to evaluate system functionality and navigation performance. Our system was initially developed on a laptop and later deployed on an edge computing device. Figure 4 shows the wearable setup of our navigation system for visually impaired users. It was a head-mounted design that included an Intel RealSense D455 depth camera (Intel Corporation, Santa Clara, CA, USA), a 3D-printed bracket, headphones, and a laptop. The bracket worked with standard GoPro head straps. It provided a stable fit, securely supported the sensors, and enabled real-time navigation testing. The laptop ran Ubuntu 20.04 and was equipped with an Intel i5-8250U processor, an NVIDIA GeForce MX110 graphics card (NVIDIA Corporation, Santa Clara, CA, USA), and 8 GB of RAM. ROS Noetic was used as the middleware to manage communication and synchronization between modules. Our system was mainly developed in Python (version 3.8.0). It used RTAB-Map for localization and map building, YOLO-World for object detection, GPT-4o-mini for semantic generation, Python package SpeechRecognition for voice input, and Edge-TTS for speech synthesis and output.

### 3.4. Hallucination Evaluation

For hallucination evaluation, a benchmark dataset was used. Experiments were conducted on the revised polling-based object probing evaluation (RePOPE) dataset [26]. This evaluation assessed the system’s effectiveness in reducing hallucinations in vision-language tasks. RePOPE was designed to examine object hallucination, a phenomenon in which models generated objects that did not exist in the image while responding to image-based questions.

RePOPE was a revised version of the POPE [27] dataset. It corrected and refined annotation errors in the MS COCO 2014 validation set [28] to better capture the hallucination behavior of VLMs. The core idea was that if a model described an object that was not present in the image, it was considered a hallucination error. The dataset included 500 images, each annotated with at least three objects. Six yes/no questions were created for each image, resulting in about 9000 Q&A pairs. The questions were divided equally into positive samples and negative samples. Negative samples included co-occurring objects designed to trigger hallucination errors. For example, our system may ask about a “dog” when only a “person” was present. This setup simulated cases where the model was influenced by linguistic bias or co-occurrence patterns. The data were stored in JSON format. Each entry included the image filename, the question text (e.g., “Is there a person in the image?”), and a label (“yes” or “no”).

**Figure 5:**
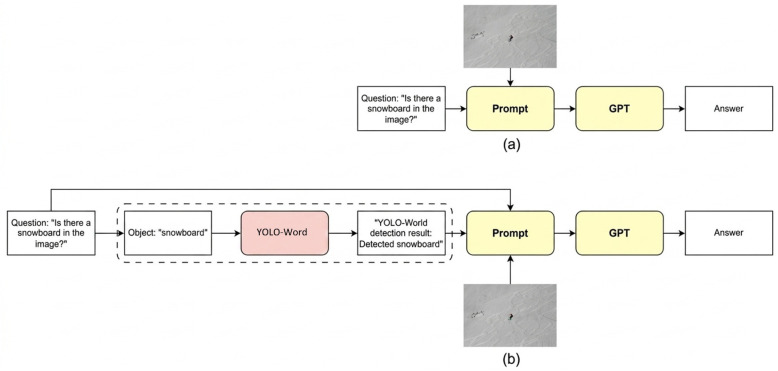
Comparison of the traditional VLM and the proposed approach for reducing hallucinations. (**a**) The traditional VLM method; (**b**) The proposed approach.

To verify system performance, we compared two model architectures. The baseline was the traditional VLM architecture, GPT-4o-mini, where images and questions were directly input into a language model to generate answers. The other was the proposed method, denoted as GPT + YOLO, integrating a YOLO-World perception component. We first extracted object keywords (e.g., “snowboard”) from the question and performed detection. If the object was detected, the result (e.g., “Detected snowboard”) was added to the prompt before being sent to the language model. Evaluation metrics included accuracy, precision, recall, and F1-score under different sampling settings. Figure 5 shows a comparison between the traditional VLM and the proposed approach for reducing hallucinations.

### 3.5. Real-World Navigation

Experiments were conducted in a campus building with several representative semantic spaces, including “restrooms,” “stairs,” “laboratories,” and “elevator area.” After constructing the map, semantic labels were manually added and converted into an OSMAG topological graph for semantic path planning. Navigation tasks were designed to evaluate both single-floor and multi-floor applications. For each navigation task, the system first generated a semantic path based on the topological map. Real-time perception and language reasoning were then used to produce step-by-step guidance. The user followed the generated instructions to reach the target location. System response time and navigation consistency were recorded.

The actual navigation trajectories were recorded using the RTAB-Map localization system, which provided continuous pose estimation during navigation. The recorded trajectories were compared with the planned semantic paths to assess navigation consistency and correctness. System performance was evaluated using both quantitative and qualitative metrics. Response time was measured as the duration between user input and system output. Navigation performance was assessed by comparing planned paths with actual trajectories and analyzing deviations and consistency.

## 4. Results

We first evaluated the system using a benchmark dataset to assess hallucination reduction, followed by real-world navigation experiments in indoor environments to validate practical performance.

### 4.1. Hallucination Evaluation Results

Experiments were conducted under three sampling strategies: adversarial, popular, and random. Table 1 summarizes the performance comparison between the baseline model (GPT-4o-mini) and the proposed GPT + YOLO approach. Precision increased by 1.55%, 2.29%, and 2.17% under adversarial, random, and popular settings, respectively. Recall increased slightly across all settings, while the F1-score showed consistent gains ranging from 0.68% to 1.23%.

### 4.2. Real-World Navigation Results

Two real-world cases were designed to evaluate our system under different task requirements. Each case included five aspects: shortest path planning, semantic path generation, path planning visualization, actual movement trajectory, and guidance instructions.

(1) Case 1: This case demonstrated the proposed system workflow and the accuracy of the voice guidance function. No obstacles were placed on the path from the starting point to the target. The user followed the voice prompts and visual guidance to reach the destination. This setting evaluated module collaboration, navigation accuracy, and system operation.

The starting point of this case was Room 1421 on the 14th floor, and the destination was the Men’s Restroom. Based on the topological semantic map, the shortest path was generated as: {“path”: [“1421”, “1422”, “1423”, “1424”, “Men’s Restroom”]}. The semantic path generation module then converted this path into clear natural language steps:

(1)  Start at “1421”.
(2)  Walk to “1422”.
(3)  Continue to “1423”.
(4)  Then walk to “1424”.
(5)  Finally, enter the “Men’s Restroom”.

**Figure 6:**
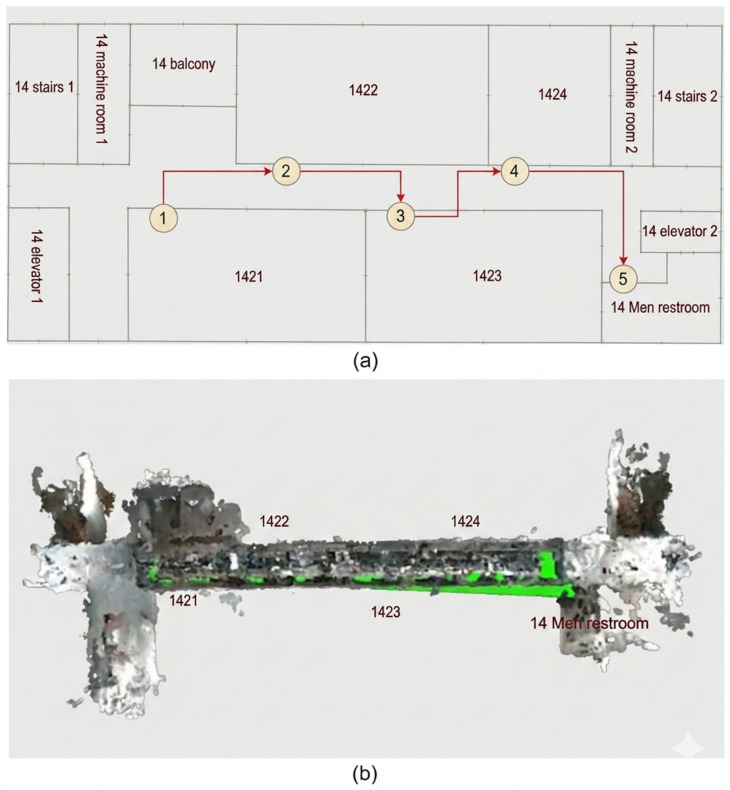
Performance evaluation under Case 1. (**a**) Planned paths generated by the navigation algorithm; (**b**) actual trajectories recorded during the real-world navigation experiments.

The semantic description included intermediate nodes, direction cues, and scene-specific location descriptions to help the user understand the path. Figure 6 shows the path planning results and the actual movement trajectories. The recorded trajectories matched the planned paths during navigation. The pauses and turns at each path node matched the expected route, showing consistency between our system prompts and the user’s actions. We recorded four guidance instances and measured their response times. The mean computation time across the four cases was 3.286 s. The guidance messages combined current node information with simple scene descriptions. The instructions were clear and structured, helping users understand the environment and follow the correct navigation actions.

(2) Case 2: This case simulated a visually impaired user navigating across multiple floors. The goal was to test our system’s semantic reasoning and guidance when the user encountered non-flat areas, such as stairs. The navigation started from Room 1421 on the 14th floor and targeted Room 1521 on the 15th floor. Based on the constructed topological semantic map, the shortest route was expressed as {“path”: [“1421”, “14 elevator 1”, “14 stairs 1”, “15 stairs 1”, “15 elevator 1”, “1521”]}. This semantic path was further translated into a multi-step natural language guide:

(1)  Start from “Room 1421” on the 14th floor.
(2)  Walk to “14 elevator 1” and pass it because it is out of order.
(3)  Continue to “14 stairs 1”.
(4)  Use the stairs to go to the 15th floor and arrive at “15 stairs 1”.
(5)  Walk to “15 elevator 1” and pass it.
(6)  Finally, arrive at “Room 1521”.

**Figure 7:**
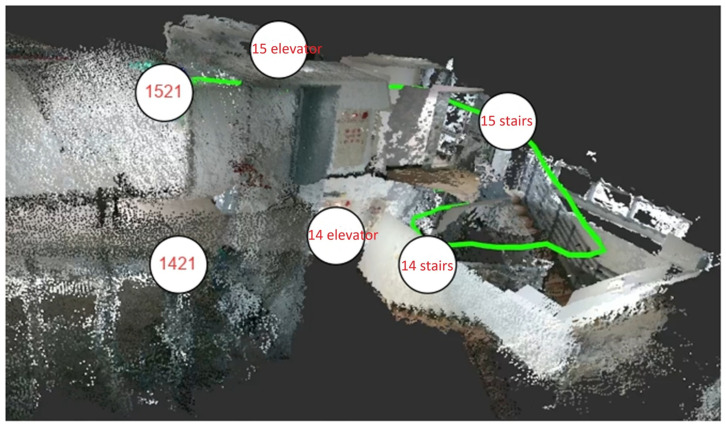
Performance evaluation for Case 2. Actual movement trajectories corresponding to the path planning results shown in Figure 3.

The generated path avoided nonfunctional elevators. Figure 7 illustrates the actual movement trajectories derived from the path planning results depicted in Figure 3. The user completed the inter-floor navigation task by following our system’s voice guidance. The guidance time was 7.247 s.

## 5. Discussion

The experimental results demonstrated that the proposed system achieved consistent improvements over the baseline VLM across all evaluation settings. The observed gains in precision indicated that integrating perception-grounded semantic reasoning effectively reduced hallucination by anchoring language generation to reliable visual evidence. The stable recall further suggested that the improvement in prediction reliability was achieved without compromising detection sensitivity. Moreover, the improved performance under adversarial conditions highlighted the robustness of the proposed approach in mitigating semantic bias and handling misleading object co-occurrence patterns.

Compared with conventional approaches, the proposed system provided a unified framework integrating perception, mapping, and language interaction. This allowed context-aware and explainable navigation guidance, which was particularly beneficial in complex environments. For instance, in stair navigation applications, the proposed system generated safety-aware instructions that incorporated spatial structure and environmental cues, demonstrating its ability to adapt to semantically rich conditions.

Despite these advantages, several limitations remained. First, the system relied heavily on perception accuracy. AI misinterpretation of the environment, including false positives, missed objects, or incorrect semantic understanding, may directly affect the correctness of navigation instructions and introduce potential safety risks, such as missed obstacle detection or misleading guidance. To mitigate these risks, the proposed system integrated perception-grounded reasoning, multimodal information fusion, and safety-aware instruction design. Second, system latency remained a challenge in real-time applications. Increased reasoning complexity and unstable wireless connectivity, particularly in environments such as stairwells, can lead to delayed responses. Third, the current system depended on manually constructed semantic maps, which limited scalability in large-scale or dynamic environments. Finally, the experimental evaluation was conducted without visually impaired participants, which restricted the assessment of usability in real-world assistive applications.

To address these limitations, future work will focus on several directions. To mitigate the potential safety risks, further enhancing perception robustness through multi-sensor fusion, temporal consistency modeling, and uncertainty estimation may improve overall system reliability. Reducing latency was essential for real-time performance. This can be achieved through lightweight models, on-device inference, and hybrid cloud–edge architectures. In addition, automated semantic mapping techniques, such as semantic SLAM and vision-language-based scene understanding, will be explored to support scalable real-world applications. Furthermore, user-centered studies involving visually impaired participants will be conducted to evaluate usability, cognitive load, and interaction effectiveness. Adaptive guidance strategies, including personalized instruction levels and context-aware simplification, will also be investigated to improve user experience and acceptance.

## 6. Conclusions

We have presented a multimodal navigation system that integrates perception, semantic mapping, and vision-language reasoning to assist visually impaired users in indoor environments. The proposed approach improves navigation performance and reduces hallucination by grounding language generation in real-time perception. Experimental results from benchmark evaluation and real-world environments demonstrate the effectiveness and feasibility of the system. Overall, the proposed system highlights the potential of integrating perception and language models for assistive navigation and provides a foundation for future research on scalable, user-centered, and real-time intelligent guidance systems. Hence, perception-grounded multimodal reasoning is a promising direction for developing reliable and intelligent assistive navigation technologies.

## Figures and Tables

**Table 1:** Performance comparison on the RePOPE dataset. The best results were shown in bold.

Setting	Model	Accuracy (%)	Precision (%)	Recall (%)	F1-Score (%)
Adversarial	GPT-4o-mini	87.22	92.97	76.92	84.19
	GPT + YOLO	**87.85**	**94.52**	**77.00**	**84.87**
Random	GPT-4o-mini	89.87	96.21	78.86	86.68
	GPT + YOLO	**90.88**	**98.50**	**79.38**	**87.91**
Popular	GPT-4o-mini	87.09	93.22	76.03	83.75
	GPT + YOLO	**88.01**	**95.39**	**76.28**	**84.77**

## Data Availability

The data presented in this study are available upon request from the authors.

## Abbreviations

The following abbreviations are used in this manuscript:

AI	Artificial Intelligence
BFS	Breadth-First Search
GPT	Generative Pre-trained Transformers
IMU	Inertial Measurement Unit
LiDAR	Light Detection and Ranging
LLM	Large Language Model
OSMAG	Open Semantic Map and Graph
Q&A	Question and Answer
RePOPE	Revised Polling-based Object Probing Evaluation
RFID	Radio Frequency Identification
ROS	Robot Operating System
RTAB-Map	Real-Time Appearance-Based Mapping
SLAM	Simultaneous Localization and Mapping
TTS	Text-to-Speech
UWB	Ultra-Wideband
VLM	Vision-Language Model
VQA	Visual Question Answering
YOLO	You Only Look Once
